# Supplementary material for: Continental scale dietary patterns in a New World raptor using web-sourced photographs
Source: PLoS One. 2024 Jul 15;19(7):e0304740. doi: 10.1371/journal.pone.0304740 (PMC11249219; doi:10.1371/journal.pone.0304740)
Supplement: S1 Table — ‘GLM’ = Generalized Linear Model, ‘GLMM’ = Generalized Linear Mixed Model. (DOCX) [file pone.0304740.s001.docx]

**Table S1.** An overview of the statistical modeling process undertaken to explore the effects of age, population and latitude on the diet of Crested Caracaras (*Caracara plancus*) throughout North, Central and South America between 1987 through 2022. ‘GLM’ = Generalized Linear Model, ‘GLMM’ = Generalized Linear Mixed Model.

| Model number | Model type | Data set | Model terms (variable type) | | | Data family | Error distribution |
| --- | --- | --- | --- | --- | --- | --- | --- |
|  |  |  | Response | Explanatory | Random |  |  |
| *Food group, age and population model* | | | |  |  |  |  |
| 1 | Multinomial log-linear model | full | food group (categorical) | age (categorical) | - | - | - |
|  |  |  |  | population (categorical) |  |  |  |
|  |  |  |  | age × population |  |  |  |
| *Food group, latitude and population models* | | | |  |  |  |  |
| 2 | GLM | northern | invertebrate (1,0 binary) | latitude (continuous) | - | binomial | logit |
| 3 | GLMM | northern | bird (1,0 binary) | latitude (continuous) | cluster ID (categorical) | binomial | logit |
| 4 | GLMM | northern | fish (1,0 binary) | latitude (continuous) | cluster ID (categorical) | binomial | logit |
| 5* | GLMM | northern | garbage (1,0 binary) | latitude (continuous) | cluster ID (categorical) | binomial | logit |
| 6 | GLMM | northern | mammal (1,0 binary) | latitude (continuous) | cluster ID (categorical) | binomial | logit |
| 7 | GLMM | northern | reptile (1,0 binary) | latitude (continuous) | cluster ID (categorical) | binomial | logit |
| 8 | GLMM | southern | bird (1,0 binary) | latitude (continuous) | cluster ID (categorical) | binomial | logit |
| 9 | GLMM | southern | fish (1,0 binary) | latitude (continuous) | cluster ID (categorical) | binomial | logit |
| 10 | GLMM | southern | invertebrate (1,0 binary) | latitude (continuous) | cluster ID (categorical) | binomial | logit |
| 11 | GLMM | southern | mammal (1,0 binary) | latitude (continuous) | cluster ID (categorical) | binomial | logit |
| 12 | GLMM | southern | reptile (1,0 binary) | latitude (continuous) | cluster ID (categorical) | binomial | logit |
| *the "bobyqa" optimizer using the function glmerControl() from the R package lme4 (Bates *et al*. 2015) was included to prevent model non-convergance. | | | | | | | |
