# Supplementary material for: Continental scale dietary patterns in a New World raptor using web-sourced photographs
Source: PLoS One. 2024 Jul 15;19(7):e0304740. doi: 10.1371/journal.pone.0304740 (PMC11249219; doi:10.1371/journal.pone.0304740)
Supplement: S2 Table — Significant contrasts (P < 0.05) highlighted in bold. (DOCX) [file pone.0304740.s002.docx]

**Table S2.** Contrasts from the multinomial log-linear model exploring the effects age, population and their interaction (age × population) on the probability of different food groups in photographs of Crested Caracaras (*Caracara plancus*) feeding throughout North, Central and South America between 1987 through 2022. Significant contrasts (*P* < 0.05) highlighted in **bold**.

| Contrast | Food group | Estimate | SE | df | *t* | *P* |
| --- | --- | --- | --- | --- | --- | --- |
| *Age contrasts* |  |  |  |  |  |  |
| adult northern - (non-adult northern) | birds | 0.055 | 0.033 | 20 | 1.67 | 0.363 |
| adult southern - (non-adult southern) | birds | 0.101 | 0.039 | - | 2.58 | 0.077 |
| adult northern - (non-adult northern) | fishes | -0.060 | 0.034 | - | -1.75 | 0.325 |
| adult southern - (non-adult southern) | fishes | -0.091 | 0.041 | - | -2.20 | 0.157 |
| adult northern - (non-adult northern) | garbage | 0.016 | 0.018 | - | 0.87 | 0.821 |
| adult southern - (non-adult southern) | garbage | 0.009 | 0.018 | - | 0.51 | 0.955 |
| adult northern - (non-adult northern) | invertebrates | -0.025 | 0.020 | - | -1.25 | 0.605 |
| adult southern - (non-adult southern) | invertebrates | -0.027 | 0.023 | - | -1.20 | 0.637 |
| adult northern - (non-adult northern) | mammals | -0.010 | 0.043 | - | -0.23 | 0.995 |
| adult southern - (non-adult southern) | mammals | -0.005 | 0.045 | - | -0.10 | 1.000 |
| adult northern - (non-adult northern) | reptiles | 0.025 | 0.027 | - | 0.92 | 0.797 |
| adult southern - (non-adult southern) | reptiles | 0.012 | 0.019 | - | 0.65 | 0.915 |
| *Population contrasts* |  |  |  |  |  |  |
| **adult northern - adult southern** | **birds** | **-0.089** | **0.026** | **20** | **-3.48** | **0.012** |
| (non-adult northern) - (non-adult southern) | birds | -0.043 | 0.044 | - | -0.97 | 0.770 |
| adult northern - adult southern | fishes | -0.050 | 0.022 | - | -2.26 | 0.141 |
| (non-adult northern) - (non-adult southern) | fishes | -0.081 | 0.049 | - | -1.65 | 0.376 |
| adult northern - adult southern | garbage | 0.016 | 0.013 | - | 1.29 | 0.577 |
| (non-adult northern) - (non-adult southern) | garbage | 0.009 | 0.022 | - | 0.43 | 0.972 |
| adult northern - adult southern | invertebrates | -0.004 | 0.011 | - | -0.36 | 0.983 |
| (non-adult northern) - (non-adult southern) | invertebrates | -0.006 | 0.028 | - | -0.21 | 0.997 |
| adult northern - adult southern | mammals | 0.050 | 0.028 | - | 1.76 | 0.319 |
| (non-adult northern) - (non-adult southern) | mammals | 0.055 | 0.056 | - | 0.99 | 0.757 |
| **adult northern - adult southern** | **reptiles** | **0.077** | **0.016** | **-** | **4.85** | **0.001** |
| (non-adult northern) - (non-adult southern) | reptiles | 0.065 | 0.029 | - | 2.22 | 0.152 |
