# Supplementary material for: Continental scale dietary patterns in a New World raptor using web-sourced photographs
Source: PLoS One. 2024 Jul 15;19(7):e0304740. doi: 10.1371/journal.pone.0304740 (PMC11249219; doi:10.1371/journal.pone.0304740)
Supplement: S3 Table — SE = standard error, df = degrees of freedom. Significant effects (P < 0.05) in bold. (DOCX) [file pone.0304740.s003.docx]

**Table S3.** Outputs from the generalized linear mixed models exploring the effect of latitude on the probabilities of different food groups within photographs of Crested Caracaras (*Caracara plancus*) feeding throughout North, Central and North America. SE = standard error, df = degrees of freedom. Significant effects (*P* < 0.05) in **bold**.

| Food group | Estimate | SE | df | *z* | *P* |
| --- | --- | --- | --- | --- | --- |
| *Northern population* | |  |  |  |  |
| birds | -0.011 | 0.016 | 533 | -0.662 | 0.508 |
| **fishes** | **-0.044** | **0.020** | - | **-2.251** | **0.024** |
| garbage | -0.017 | 0.029 | **-** | -0.601 | 0.548 |
| **mammals** | **0.060** | **0.015** | **-** | **3.974** | **< 0.0001** |
| reptiles | -0.015 | 0.028 | **-** | -0.539 | 0.590 |
| *Southern population* | |  |  |  |  |
| birds | 0.010 | 0.019 | 376 | 0.539 | 0.590 |
| **fishes** | **0.061** | **0.025** | - | **2.467** | **0.014** |
| invertebrates | 0.042 | 0.033 | - | 1.267 | 0.205 |
| **mammals** | **-0.072** | **0.016** | - | **-4.432** | **< 0.0001** |
| reptiles | 0.053 | 0.032 | **-** | 1.636 | 0.102 |
