# Supplementary material for: Continental scale dietary patterns in a New World raptor using web-sourced photographs
Source: PLoS One. 2024 Jul 15;19(7):e0304740. doi: 10.1371/journal.pone.0304740 (PMC11249219; doi:10.1371/journal.pone.0304740)
Supplement: S4 Table — SE = standard error, df = degrees of freedom. Significant effect (P < 0.05) in bold. (DOCX) [file pone.0304740.s004.docx]

**Table S4.** Outputs from the generalized linear model exploring the effect of latitude on the probability of invertebrates within photographs of Crested Caracaras (*Caracara plancus*), from the northern population only, feeding throughout North, Central and North America. SE = standard error, df = degrees of freedom. Significant effect (*P* < 0.05) in **bold**.

| Food group | Estimate | SE | df | *z* | *P* |
| --- | --- | --- | --- | --- | --- |
| *Northern population* | |  |  |  |  |
| **invertebrates** | **-0.084** | **0.018** | 918 | **-4.616** | **< 0.0001** |
