# Supplementary material for: Continental scale dietary patterns in a New World raptor using web-sourced photographs
Source: PLoS One. 2024 Jul 15;19(7):e0304740. doi: 10.1371/journal.pone.0304740 (PMC11249219; doi:10.1371/journal.pone.0304740)
Supplement: Attachment — Submitted filename: Miranda_PONE-D-23-42552.docx [file pone.0304740.s005.docx]

The present study utilizes an online dataset as a tool to acquire knowledge about the caracara diet. The researchers employed robust statistics and large sample sizes to test various hypotheses regarding caracara foraging. They exhibited wit and precision in their test choices and interpretations. Based on their observations, the authors infer that the caracara diet remains consistent with existing descriptions; the species is a known generalist. However, the proof of concept they present allows for the application of the same methods to eagles or other predators for which dietary data might be both crucial and scarce—a point that warrants discussion. Additionally, other observed diet patterns strongly align with their associated data. This study, both simple and robust, epitomizes citizen-based science in my view. I believe PLOS ONE is a suitable publication venue for this manuscript and I wholeheartedly recommend it for publication. Congratulations.

L52: Please rephrase this section since it's not about a misinterpretation but rather a misunderstanding of the natural world.

L66-77: This trend extends to other predators like giant snakes and carnivores, among others.

L77-80: In essence, this provides an opportunity to study a species that have been extensively researched; which is no opportunity at all. Please note that the methods mentioned and criticized in the beginning of the previous paragraph aren't limited to easy species. It feels like cherry picking from your side in favour of your collection method; an effort to address this would enhance the text. Moreover, it makes the argument over the selection of your study species somewhat circular. None of this, in my opinion, is detrimental to the paper, but the text should be revised—especially concerning the choice of the study species.

L94: It surprises me that you chose to overlook larger databases such as WikiAves or iNaturalist. Could you provide some background on your choice?

L108-237: I'm impressed by the clarity, elegance, and robustness of the methods and sample design. The threshold sample sizes for dietary analysis were particularly impressive. However, the categorization of prey and other food sources feels somewhat artificial to me. I believe including functional groups—like domestic animals or aquatic species? L225-228—might make more sense. Nevertheless, congratulations on the excellent work.

L257: I'm absolutely impressed by the broad and extensive sample. I'd like the study to explore a rarer, threatened, or less-known species. However, this doesn't diminish the outstanding work you've done.
