# Supplementary material for: Continental scale dietary patterns in a New World raptor using web-sourced photographs
Source: PLoS One. 2024 Jul 15;19(7):e0304740. doi: 10.1371/journal.pone.0304740 (PMC11249219; doi:10.1371/journal.pone.0304740)
Supplement: Attachment — Submitted filename: Response to reviewers PONE-D-23-42552-R1 (2).docx [file pone.0304740.s006.docx]

**Response to reviewers PONE-D-23-42552**

**Comments from editor:**

Your study is very interesting and highlights that citizen science data (photographs) can be used to investigate dietary aspects of birds at large spatial and temporal scales in the Neotropics. The approach is quite appropriate and the results are interesting.

I´m mainly concerned with the restriction of your sample to data from the Macaulay Library (that encompasses eBird). For example, the WikiAves platform (<https://www.wikiaves.com.br>) harbors a lot of data about Brazilian birds, including 27,100 records of Caracara plancus. On 08 January 2024, I clicked on “Registros” (Records), and then on “Busca Avançada” (Advanced search). Then, I typed the name of the species (“Espécie”, Species) and then, near the page bottom, clicked on the option “Alimentando-se/caçando” (Feeding/hunting). This search resulted in 3,429 photographs of C. plancus. Of these, about 50% might have evidence of feeding activities by this raptor in Brazil (I guess this percentage based on studies that I have done using this platform – e.g., Rupornis magnirostris). This available amount of records would represent a good sample of food items along a considerable latitudinal range, in your study. Another option with less numerous records for South America would be iNaturalist….these two additional searches could double your sample size, or do even more….please think about (R1 and R2 also pointed out this question). The current sample could lead to negative criticism by readers, despite the large number of records/photographs. On other hand, your results appear to be quite robust (convincing), and thus your Macaulay-based sample would be enough. Please try to del with this aspect in the Methods and Discussion sections.

>>> Thank you for taking the time to consider our manuscript, and for your positive and constructive review. During the revision process, we explored whether including additional photographs from WikiAves would be suitable for our study. We decided that due to the photographs being restricted geographically to Brazil, that this may introduce a spatial bias within our continental scale dietary assessment. Despite this, we did indeed increase our sample size by collating and processing photographs of caracaras from iNaturalist. Overall, we processed an additional 35,571 photographs from iNaturalist and together with our sample from Macaulay increased our initial sample size to 78,059. From the new photographs, after screening and removing duplicates, we added some 925 new photos to our analyses, resulting in a data set comprising 1,555 photographs used in the statistical analyses. Interestingly, our results remained largely the same when including the additional photographs. We increased our sample size for caracaras from the southern population substantially which enabled us to explore latitudinal effects in food groups that before had insufficient sample sizes. We hope that the editor agrees with us that our manuscript is much more robust compared to the initial submission.

Title – we have revised the title to shorten it and to focus on our ability to assess diet at the continental scale using our approach.

“Continental scale dietary patterns in a New World raptor using web-sourced photographs” (L1-2).

Abstract.
Ok.

>>> the abstract has been updated given the new results from the inclusion of iNaturalist data (L26; L30).

Introduction.
Very well structured with relevant publications. But note that you provided few sentences regarding latitudinal variation in the diet of birds, including raptors. For example, along lines 70-80 you can briefly present the major results of these investigations at large spatial scales. After the current examples, you could add 2-3 examples mainly focusing latitudinal variation. Also, try to reduce the amount of information on the methods relative to diet of birds. The reading is running well, just need to do these modifications for a better suitability to PLOS ONE.

>>> Thank you for these suggestions, we have expanded on latitudinal variation within bird and raptor diets in the suggested section while trying to also introduce the use of web-sourced photography as a tool to study diet. Upon reflection, we felt that this was appropriate at this point in the manuscript and provides a link to the following paragraph:

“For species that inhabitant large geographical areas, exploring how diet varies latitudinally may improve understanding of its ecology. For example, ecological and climatic conditions along latitudinal gradients influence the availability and presence of prey species within the wider environment [24], affecting the diversity and dietary composition for many species including birds [25]. Reviews of the raptor diet literature found distinct latitudinal patterns in the proportions of prey groups in some raptor diets, with increased probabilities of mammalian prey at higher latitudes [26,25].” (L68-75).

Line 51. Is there a word better than “however” ?
>>> To better emphasise the point that dietary assessments are dependent on the method and scale used, we have replaced the word “however” with “Most importantly” (L50).

Line 90. “it how diet” is correct ?
>>> Thank you for spotting this, we have now removed “it” from the sentence.

Objectives. Ok.
>>> No action required.

Material and Methods
A Study Area section would be welcome. Please try to explain main aspects of the Americas that are pertinent to your study, such as North, Central, and South Americas, maximum values of Lat in both Hemispheres, the line of Equator position. It would be useful for readers from other parts of the world.

>>> We have now revised the Methods accordingly and included a “Study Areas” subsection outlining the geographic extent of our study areas, regions and biogeographic subrealms:

## “**Study Areas**

Our study area encompasses the caracara’s entire geographic range, spanning a latitudinal gradient from approximately 45°N to -55°S, and a longitudinal gradient from approximately -35°E to -125°W. Photographic samples span multiple regions including the Nearctic of North America, Mexican Transition Zone through North/Central America and into the Neotropical zone in Central/South America [53]. According to the Bioregions 2023 Framework (<https://www.oneearth.org/bioregions/>), North American photographs represent samples from numerous biogeographic subrealms including the North Pacific Coast, American West, Mexican Drylands, Great Plains and the Southeast U.S. Savannas and Forests. Photographs of caracaras from Central America include the Central America and Caribbean biogeographic subrealms, with those from South America spanning the Andes and Pacific Coast, Upper South America, Amazonia, Brazilian Cerrado and Atlantic Coast and the South American Grassland subrealms.” (L120-132).

Section “Web-sourced…”.
If you decide to keep only with the Macaulay Library data, you will have to explain why you did not included searches in the WikiAves, iNaturalist and other databases. Like tell that you sample is large, huge, when compared with those of previous studies. On the other hand, if you decide to include other databases, then add text to explain how you did the searches.
>>> We have now included additional data from iNaturalist, please see response to comment above relating to this.

Line 116. You repeated…why not use “For them….” ?

>>> ‘useable’ now replaced with ‘For them’ (L142-143).

Line 117. What did you consider (adult or non-adult) when you could see an adult with a prey near a nestling in the nest ? This type of photograph has your two types of caracaras….

>>> The editor raises a very good point here relating to photographs where more than one bird (and age group) were visible in the photograph. We believe this was only the case for a handful of photographs. When this occurred, we only extracted age data from the bird that was interacting with the food item. We have clarified this in-text to benefit the reader

“For photographs that contained more than one caracara of each age group, we only extracted data for the individual interacting with the prey item” (L145-147).

Line 188. I did not understand item 4 (location)…can you briefly explain within brackets ?

>>> We assume the editor refers to “location” on line 120 of the original submission and not line 188. By location we refer to the surrounding features of the environment within the wider photograph, e.g., type of fencing present, road signs, etc. We have revised the text to clarify this point:

“visual characteristics or landscape features within the photograph…” (L148-149).

Line 123. These brackets are too distant. Do you really need them ? Six lines within them…can you try to reduce this text (Lines 121-130)?

>>> We agree with the editor here that the use of brackets is not appropriate for the text. As such, we have removed the brackets and streamlined the text within the paragraph:

“However, this was not always the case as there were occasions where multiple recordists uploaded images of the same feeding event across different days. This was particularly the case if the food item was large in size and thus took longer to decompose. We were able to ascertain whether these were duplicates by making an assessment based on the spatial location of the feeding event, evidence of the food item included and features of the surrounding location in the photograph.” (L152-157).

Section “Prey and age”.
Line 141, if at least two authors, than should be more than CP and FB…please check the writing.

>>> Thank you for highlighting this. We have revised to:

“visually assessed by at least two of the authors (C.P., F.B. or V.N.).” (L170).

Line 142. Better if you start a new paragraph with “Adult caracaras…”.

>>> New paragraph created as suggested (L172).

Next lines. It is not clear if you included nestlings or not. It appears that you included only juveniles outside nests, and adults. But some photographs might show 1-2 adults in nests with prey and nestlings, as I could note in WikiAves. Please clarify it.

>>>

Section “Population”.
Lines 152 and 154. Extended or extends?

>>> “extended” replaced with “extends” (L182).

You could divide this section in 2-3 paragraphs. It is too long.

>>> New paragraph created after “In an attempt…” (L199).

Calling a figure with a map would be welcome here.
>>> We have cited Figure 1 here, which show the geographic spread of the data set graphically and in a map format:

“We plotted all useable photographs in QGIS version 3.14.16 [59] and conducted an overlap analysis between the geo-referenced photograph data points and each population range polygon (Fig. 1a-b).” (LXX).

Section “statistical Analysis”
How did you control for potential influence of seasonality on these results ? For example, you might have more photographs in the rainy or dry season for a given caracara population… please note that Reviewer 1 has concern about this, or similar potential influence on data.

>>> The editor raises an interesting point here in relation to temporal differences in caracara diet. There are certainly challenges associated with studying temporal changes in raptor diets across both the northern and southern hemisphere, however, it is possible with some additional analyses. However, we believe that an additional temporal analysis would be beyond the scope of the current study given multiple aspects of the species’ diet presented here, e.g., 1) overall diet composition, 2) age effects on diet and 3) diet variation across a latitudinal gradient. However, this certainly warrants further investigation in the form of an additional study where we have the space and opportunity to conduct a more thorough assessment of the species’ diet throughout the year, without being restricted by other results presented here.

Line 238. You have to place Table 1 here.

>>> Table 1 has been moved to the position suggested by the editor (L271).

Results.
Line 244. As you write “Of these…”, this percentage would be 1501/2454). No ?

>>> Absolutely and thank you for picking this up. We have revised the percentage to “68.9%” based on 2381/3451*100. (L286-287).

Lines 250. You have to call “(Fig 1)”, abbreviated with no dot.

>>> Revised throughout manuscript.

In the end of this paragraph (Line 258), you have to bring the captions of Fig 1 and Fig 2 (but not the figures, that should be kept in the end of the manuscript). Please check Instructions again.

>>> Full figures positioned at the end of the manuscript, captions in-text (L303-322).

Line 277. Bring Table 2 here.

>>> There is no Table 2 within the main text of the manuscript.

Line 287. Bring here: Caption of figure 3…
>>> Caption for Fig 3 placed in text where suggested (L351-355).

Discussion
It would be easier to read if you use subtitles for sections of the Discussion.

>>> Thank you for this suggestion, we have now included the following subtitles to help guide the reader through the Discussion section:

1. Crested Caracara diet varied along latitudinal gradients (L367).
2. Comparisons between other diet studies (L386).
3. Web-sourced photography is a useful tool to study raptor diets across large spatial scales (L402-403).
4. Study Limitations (L424).
5. Utilizing citizen science data can be a time- and cost-effective method to study raptor diets (L452-453).
6. Conclusions (L471).

Make sure that you discuss more extensively with previous study on the diet of the species, influence or not of age among raptors, influence of latitude on raptor diets. Your current discussion is a bit poor relative to these topics. Try to the emphasis on methodology.
Always consider an international readership by citing studies conducted in several ecoregions, countries, involving several species.

>>> We have now discussed our results in line with similarly, previous research on other raptor species:

“For example, a global examination of Western Barn Owl (Tyto alba) and American Barn Owl (*T. furcata*) diets found a positive relationship in the proportion of mammal prey in colder environments [25]. Unlike previous studies which studied the diets of Eurasian Sparrowhawk and Martial Eagles [27,29], we did not find any age effects in the diet of caracaras which may be explained by their generalist nature and tendency to feed on carrion [47,66]. Eurasian Sparrowhawk are avian specialists [27,28] and age-related differences in diet may be more pronounced. Similar to our findings, a continental assessment of Montagu’s Harrier (*Circus pygargus*) diet found that mammalian prey increased at higher latitudes due to the prevalence of agricultural land cover at more northern latitudes [49].” (L408-417).

Both reviewers made some comments that might be useful for you here in the Discussion. For example, R2 commented that this study can be considered an example for the study less common, or more “difficult” species.
>>> This is an excellent point, which we agree with fully. As such, we have revised the text to reflect the comments from the editor and reviewer 2:

“Assuming that the study species is well-sampled, this approach can be applied to many raptor species, especially those that are less common or more challenging to study in the field,” (L466-468).

References.
The correct is to use like this “2. Smith TC….” Instead, you used “[2] Smith TC….”.

>>> All reference numbers have been revised following the comments from the editor.

Page numbers. There is no space before them (after the volume and :).

>>> Spaces between colon and page numbers have been removed for all relevant references.

Page number. You have to use a long dash between then, not an hifen (-).

>>> All hyphens between page numbers have now been replaced with em dashes.

DOIs. You have to provide it for all references that have it. Follow the format: (<http://doi.org/10>.....).

>>> Revised.

Ref 6. The initials in capitals should not occur, except for the first word and names….check for all.

>>> We have checked for this issue in all references and included lower cases where appropriate in references 6 and 7.

Ref 26. It occurs with a duplicate. You will need to change all these numbers here and in the text.

>>> Now revised.

Please also check Instructions for books, chapters, websites….
>>> Checked.

Tables.
They should be moved to the text, by the end of the paragraph that cite them.
>>> As requested, tables have been moved in text following previous suggestions from the editor.

Figures.
Their captions should be in the text, as above for tables. Keep figures here in the end with their “Fig x”.

>>> As requested, figure captions have been moved in-text and the figures placed at the end of the manuscript with only the Figure number provided.

Fig 1. Can you increase a bit the size of letters and numbers ? Maybe, titles of axes in bold. Is “divide” correct ? (along the dashed line).
>>> Now revised.

Fig 2. You used a duck, but do caracaras prey on them ? What about a pigeon or other smaller bird ?
>>> We have replaced this with a seabird, as there were numerous within our data set.

**Reviewer 1 comments:**

I thank the editor for inviting me to review this manuscript. The authors have presented the background, methods, results and discussion in a technically sound manner. I have a few suggestions for some of the sections which I believe will help add more clarity to the manuscript. The detailed comments are as follows:

>>> Thank you for taking the time to review our submission and for providing helpful comments which no doubt has improved our study.

Introduction
Line 44-45: The sentence can be rephrased – “Food availability and abundance can affect population dynamics of raptors”

>>> Sentence revised as suggested (L44-45).

Line 50: “provisioning” instead of “provision”

>>> Word revised as suggested (L50).

Line 57-59: It will be good to add some citation. Suggestion: <https://link.springer.com/article/10.1007/s10344-010-0480-z>

>>> Caro et al. (2011) added following the reviewer’s suggestion (L61).

Line 77-79: This sentence can be deleted. Information repeating in the beginning of the para.

>>> Thank you for the suggestion. We respectfully disagree about removing this sentence entirely, as it provides a link to the study species’ tendency to occupy human modified landscapes. Ending this paragraph on a short sentence about Tiny Hawks seemed a little disjointed with the next paragraph. As such, we have merged the two sentences together and shortened it to maintain flow into the next paragraph. We hope that the reviewer agrees with our choice here:

“Despite this, web-sourced photography is a useful tool to study well-sampled species that inhabitant large geographic areas including open landscapes and those associated with human activities [28,29].” (L86-89).

Line 90: “it” can be removed from the last sentence

>>> Thank you for spotting this, “it” has now been removed.

Line 91: Will be good to add information on status of the species in the Americas, especially since it occupies open habitats which are under threat globally.

>>> We have expanded on this point in the methods section where we provide information on the two populations:

“This species tends to inhabit more open landscapes where it scavenges and hunts for food, and is often observed in human-modified habitats [56].” (L185-187).

Methods
Line 111: Any justification as to why only Macaulay library was considered for collecting photographs? What about other social media databases such as Facebook, Flickr etc.?

>>> We have previously used photograph data from social media platforms such as Facebook, Flickr and Twitter. However, the algorithms constantly refresh content making it difficult for somebody to repeat our sampling strategy and obtain the same results. Furthermore, Facebook’s lack of scrolling limits results in a never ending conveyor belt of content to process. Because of this, knowing when to stop can often be subjective, therefore, we decided to focus on Macaulay Library (which is linked to eBird) for this study.

After consideration, we have also included data from iNaturalist which contains useable photographic data from the Americas – in line with comments from the editor and reviewers. Please see (L134-159) for methodological overview of this process and the updated results section.

Line 169: Which year is the latest update of this range map?

>>> We believe this range map is from the latest IUCN Red List assessment for the species which was published in 2023 (<https://www.iucnredlist.org/species/22729784/232384538>). BirdLife International works with a different taxonomic backbone and considers *Caracara plancus* as a single species. We have added the updated time scales to the manuscript text to clarify this point:

“…range maps from the BirdLife International Datazone database (<http://datazone.birdlife.org/home>; previously updated in 2023)” (L201).

Line 184: Were these data points location data? It will be good to specify for clarity.

>>> Yes, these were randomly assigned geographic data points. Please see revised text:

“First, we computed 5,000 randomly distributed geographic data points across the…” (L217).

Results
Line 261-264: Can this information also be presented in Table 1 separately? The table currently has information on 1501 photographs. But food groups could be assigned to only 1029 right?

>>> During the revision process and inclusion of iNaturalist data, we have revised this section entirely:

“A total of 78,059 photographs of caracaras, taken across the Americas between 1987 through 2022, were downloaded from Macaulay Library (N = 42,488) and iNaturalist (N = 35,571) and were visually examined. Approximately 4.4% (N = 3,451) (Macaulay Library = 2,454; iNaturalist = 997) of the photographs appeared to include caracaras feeding and were subsequently marked as ‘of interest’. Of these photographs, 68.9% (N = 2,381; Macaulay Library = 1,456; iNaturalist = 925) were classified as ‘useable’ as it was confirmed that they contained a caracara feeding. Of all useable photographs, there were a far higher proportion of adults (N = 1,948; 81.8%) than non-adults (N = 433; 18.2%) and photographs were distributed across both population ranges (Table 1) with distributions of photographs for both populations peaking between approximately ≥ 20 ≤ 29.9° (northern population) and ≥ -40 ≤ -30.1° latitude (southern population) (Fig. 1a-b). A total of 1,445 (60.7%) photographs were from the northern population and 936 (39.3%) from the southern population (Table 1). For the northern population 1,214 (84% northern population) photographs were of adults and 231 (16%) included non-adults (see Fig. 2a-f for examples). For the southern population, 734 (78.4% southern population) photographs were of adults and 202 (21.6%) included non-adults. There were 796 photographs that we were unable to assign to a broad food group, and together with the 30 photographs representing amphibian and plant food groups, these were excluded prior to the analyses. We were able to assign broad food groups to 1,555 (65.3% of useable photos) photographs which were used in the analyses (Table 1).” (L282-201). See also revised Table 1.

Line 274: This result has not been discussed in detail in the discussion section. What might be the reason for higher probability of reptiles in the northern population diet?

>>> We suspect this may be due to 1) larger sample sizes for northern birds per unit area relative to the southern population and 2) availability of suitable habitat, especially in Mexico, which tend to have increased reptile diversity compared to more temperate/boreal regions. However, we did not explicitly test this in our analyses. We have now discussed this in the Discussion:

“There was a significant increase in the probability of reptiles in the diet of caracaras from the northern population, and the opposite pattern in the probability of birds in the diet of the southern caracara population. Although we suspect this may be a confounding effect of both larger sample sizes per-unit-area when compared to the southern population and availability of suitable habitat for reptiles especially across Mexico [64]. “ (L371-376).

Discussion
Line 303-306: Will it be good to test for seasonality of occurrence of these food groups from photographs? Maybe occurrence of invertebrates in diet might be more during the warmer months in areas away from the equator. The availability of these food sources might be limited to warmer seasons in the temperate regions.

>>> This is an interesting point relating to temporal differences in caracara diet. There are certainly challenges associated with studying temporal changes in raptor diets across both the northern and southern hemisphere, however, it is possible with some additional analyses. However, we believe that an additional temporal analysis would be beyond the scope of the current study given multiple aspects of the species’ diet presented here, e.g., 1) overall diet composition, 2) age effects on diet and 3) diet variation across a latitudinal gradient. However, this certainly warrants further investigation in the form of an additional study where we have the space and opportunity to conduct a more robust assessment of the species’ diet throughout the year, without being restricted by other results presented here. Nethertheless, we would like to thank the reviewer for this excellent idea.

Lines 315-318: This reference is missing in the literature cited. I believe it’s this study: <https://doi.org/10.1006/jare.2000.0745>

>>> Thank you, we have now added this missing reference to the reference list (L645-647).

Line 329: There are two citations with the same number [26]. This has to be rectified in the references section.

>>> Thank you for spotting this, we have now checked and revised the references in sequential order.

Figures
Figure 1: Along with the latitudinal degrees, it will also be good to show the direction on the x-axis (N/S).

>>> As we did not test for the effects of longitude on caracara diet, we feel that it would be best to keep the figures as simple as possible reflecting the analyses performed (without confusing the reader). We would like to thank the reviewer for the suggestion. Please see the revised figure, which we believe is much improved.

Figure 2 a.: The lat longs can be shown in the form of a grid on the map along with the equator line. Since the major part of the study is about latitudinal variation and possible differences in diet in regions away from the equator.

>>> To avoid ambiguity between the revised panel figure 1a and 1b, we decided not to include xy coordinates alongside the map, as latitudinal categories are presented on the y-axis of Figure 1a. Please see the revised figure, which we believe is much improved.

Figure 2 b.: The “**” is not clearly visible on the graph. The font can be made larger.

>>> Asterisks now increased in size.

**Reviewer 2 comments:**

The present study utilizes an online dataset as a tool to acquire knowledge about the caracara diet. The researchers employed robust statistics and large sample sizes to test various hypotheses regarding caracara foraging. They exhibited wit and precision in their test choices and interpretations. Based on their observations, the authors infer that the caracara diet remains consistent with existing descriptions; the species is a known generalist. However, the proof of concept they present allows for the application of the same methods to eagles or other predators for which dietary data might be both crucial and scarce—a point that warrants discussion. Additionally, other observed diet patterns strongly align with their associated data. This study, both simple and robust, epitomizes citizen-based science in my view. I believe PLOS ONE is a suitable publication venue for this manuscript and I wholeheartedly recommend it for publication. Congratulations.

>>> Thank you very much for taking the time to review our manuscript, and for your support with the publication of our work. Please see below our point-by-point responses.

L52: Please rephrase this section since it's not about a misinterpretation but rather a misunderstanding of the natural world.

>>> This is an important point, in line with the reviewer’s comment, we have revised this sentence to the following:

“Most importantly, the methods used to collect data on raptor diets, and the scales at which data are collected at, may lead to a misunderstanding of natural environments due to the introduction and constraints of methodological limitations.” (L50-53).

L66-77: This trend extends to other predators like giant snakes and carnivores, among others.

>>> This is a particularly good point, and we agree that this approach is widely applicable to a range of predator species including snakes. We have incorporated this suggestion in the Discussion and not in the introduction as we had already introduced and focused on raptor ecology at this point. Please see the following in the Discussion with a relevant citation:

“Assuming that the study species is well-sampled this approach can be applied to many other predatory species, including those that are inconspicuous or more challenging to study in the field, such as snakes...” (L466-468).

L77-80: In essence, this provides an opportunity to study a species that have been extensively researched; which is no opportunity at all. Please note that the methods mentioned and criticized in the beginning of the previous paragraph aren't limited to easy species. It feels like cherry picking from your side in favour of your collection method; an effort to address this would enhance the text. Moreover, it makes the argument over the selection of your study species somewhat circular. None of this, in my opinion, is detrimental to the paper, but the text should be revised—especially concerning the choice of the study species.

>>> Thank you for this comment. Our original approach was not to critize the use of more traditional methods to study raptor diet, but to simply outline the limitations of these approaches which web-sourced photography can overcome. Upon reflection, we recognise that this was not fully communicated in the text. Therefore, we have revised this section and also emphasised this point in the Discussion section:

“However, the role of more traditional approaches to study raptor diets must not be underestimated as they provide vital data on cryptic species and those that can be challenging to study in the field, e.g., tropical forest raptors [31]. Despite this, web-sourced photography is a useful tool to study well-sampled species that inhabitant large geographic areas including open landscapes and those associated with human activities [28,29].” (L84-89).

“A key assumption when using web-sourced photography pertains to the species being well-sampled. This approach, unlike more traditional techniques, e.g., analysis of pellet remains, is unsuitable when studying cryptic species or those that are challenging to observe in the field. Therefore, strengths lie in its ability to be used in conjunction with other methods complementing future raptor research [69].” (L439-444).

L94: It surprises me that you chose to overlook larger databases such as WikiAves or iNaturalist. Could you provide some background on your choice?

>>> Initially, we decided to only use the Macaulay Library data due to the large sample sizes obtained allowing us to answer our research questions. Following comments from the editor and reviewers, we have performed additional data collection from iNaturalist (an additional ~950 photographs) to improve the robustness of our study while utilising an important data source (see L134-159; Table 1).

L108-237: I'm impressed by the clarity, elegance, and robustness of the methods and sample design. The threshold sample sizes for dietary analysis were particularly impressive. However, the categorization of prey and other food sources feels somewhat artificial to me. I believe including functional groups—like domestic animals or aquatic species? L225-228—might make more sense. Nevertheless, congratulations on the excellent work.

>>> Thank you for this suggestion. We began by collecting species-level prey data, attempting to group these by prey groups highlighted above, and soon realised that the sample sizes at the species-level were too low to conduct robust statistical analyses of the data set. Therefore, we simplified our approach and decided to categorise the prey data into the groups presented within the manuscript.

L257: I'm absolutely impressed by the broad and extensive sample. I'd like the study to explore a rarer, threatened, or less-known species. However, this doesn't diminish the outstanding work you've done.

>>> Thank you for your kind comments about our work. This is certainly an interesting idea which we would be happy to consider in a future study.

**Reviewer 3 comments:**

In the manuscript entitled: “Using web-sourced photographs to explore continental-scale dietary patterns in a New World raptor, the Crested Caracara (Caracara plancus)” the authors used web-sourced photography and explored continental-scale demographic and latitudinal dietary patterns between adult and non-adult Crested Caracaras throughout the species’ range across the Americas. The paper is well written, well structured and has valuable information given that on the one hand, there is little work of this type and even less in the southern hemisphere.

However, the manuscript, as it is written now, I consider it to be methodological in nature as the focus is on the advantages of web-sourced photographs, so it may not be of interest to a wider audience such as PlosOne.

>>> Thank you for taking the time to review our work. Respectfully, we disagree with the reviewer here in relation to the methodological focus of the manuscript. We believe that PLOS One, with its broad readership, is a suitable journal for a methods-based paper as our approach is widely applicable for other well-sampled species. If it were more species-focused, then it would be better suited to an ornithology journal. We hope the reviewer agrees with our approach in this regard. Please see our point-by-point comments below.

In addition, I have some comments that may help to improve the manuscript:

Abstract-Introduction:
In the introduction, predictions are made as to what they expected to find in terms of age, latitude, mammal consumption and taxonomic groups, however this is not indicated in the abstract.

>>> Thank you for the suggestion, however, we are restricted with what we can include in the abstract given the 250 word limit. We feel that the abstract reads well in its current form.

Line 75. Indicates that previous work examined the diet, using this approach?
>>> Yes, that study used web-sourced photography too. We have now clarified this in-text (L82).

Materials and Methods:
Línea 204-206. This information is redundant, this was already stated in the sentence above (with other words), I think it is better to remove it.

>>> This sentence has now been removed.

Results:
Line 276. Figure?
>> “Fig 2” referenced as suggested.

Discussion:

At the beginning of the introduction, they should have made reference to what they had indicated at the end of the introduction (line 98-line 101). They do so in line 300-3001.

>>> The reviewer raises a critical point here. We have now revised this paragraph to focus on the initial expectations outlined at the end of the introduction, in line with the reviewer’s comments:

“**Crested Caracara diet varied along latitudinal gradients**

Our study represents the first continental-scale quantitative diet assessment for caracaras, throughout the species’ range across the Americas. As expected, for both caracara populations the probability of mammals in photographs increased with increasing distance away from the equator. There was a significant increase in the probability of reptiles in the diet of caracaras from the northern population, and the opposite pattern in the probability of birds in the diet of the southern caracara population. Although we suspect this may be a confounding effect of both larger sample sizes per-unit-area when compared to the southern population and availability of suitable habitat for reptiles especially across Mexico [64]. Contrary to patterns relating to global climatic and species diversity patterns along latitudinal gradients [50-52], the probability of reptiles showed no significant increase towards the equator for either population. However, there were significant latitudinal effects in the probabilities of fishes and invertebrates in photographs of northern caracaras, increasing towards the equator. These patterns may be driven by increased species richness within these groups towards the equator, and thus a presumed increase in availability in tropical/subtropical environments relative to temperate zones [65]. However, more research is needed to decipher these patterns.” (L367-384).

In general, the discussion is focused on the methodology, therefore, although it is correct, I consider that it would not be of interest to a wide audience and an Ornithological journal would be more appropriate for this interesting work.

>>> Thank you for your comment, please see our response above to this point.
